# Moderating Effects of Parenting Stress and COVID-19 Pandemic Impacts on Relations Between Harsh Discipline and Child Behavior Problems

**DOI:** 10.1007/s10826-025-03068-1

**Published:** 2025-05-03

**Authors:** Kivilcim Degirmencioglu, Jianing Sun, Klaudia I. Kulawska, Fanwen Zhang, Catherine M. Diercks, Erika Lunkenheimer

**Affiliations:** 1 The Pennsylvania State University, University Park, PA USA; 2 Lehigh University, Bethlehem, PA USA; 3 University of Connecticut, Storrs, CT USA; 4 WestEd, San Francisco, CA USA

**Keywords:** COVID-19 pandemic, Harsh discipline, Parenting stress, Internalizing problems, Externalizing problems

## Abstract

The present study examined the dual moderating effects of parenting stress and negative COVID-19 pandemic impacts on the link between parental harsh discipline and child behavior problems. Mothers and children aged 2–6 years (*M* = 49.68 months, 51.03% female; *N* = 339) in the United States participated online via Amazon Mechanical Turk during the COVID-19 pandemic (Summer or Winter 2020). Mothers reported on harsh discipline, parenting stress, negative COVID-19 impacts, and children’s internalizing and externalizing problems. As hypothesized, results showed a significant three-way interaction effect such that parenting stress and negative COVID-19 impacts exacerbated the positive relation between harsh discipline and child behavior problems, however, only for internalizing problems. Children had the highest levels of internalizing problems when harsh discipline, parenting stress, and negative COVID-19 impacts were higher; additionally, parenting stress still exacerbated the positive relation between harsh discipline and internalizing when pandemic impacts were lower. For externalizing problems, two-way interaction results revealed that positive relations between harsh discipline and externalizing were weaker when pandemic impacts were higher, suggesting that pandemic stressors altered these well-established effects. Findings suggested that proximal familial risks and broader environmental risks interact in complex ways to influence children’s mental health difficulties, and that interventions to reduce parenting stress may ameliorate children’s internalizing problems, especially when additional environmental stressors are present.

## Highlights

- Mothers reported the highest levels of internalizing problems for children when harsh discipline, parenting stress, and negative pandemic impacts were elevated.
- Higher parenting stress intensified the link between harsh discipline and child internalizing problems.
- Interventions addressing parenting stress may mitigate children’s internalizing problems in the context of heightened environmental risk factors, such as those associated with the COVID-19 pandemic.

In the context of the COVID-19 pandemic, families experienced significant hardships including but not limited to changes in family routines, financial difficulties, and lower social support (Brown et al., 2020). Alongside these challenges, research has also shown an increase in parenting stress and harsh discipline behaviors associated with the COVID-19 pandemic (Sari et al., 2022), underscoring its heightened risks to children’s mental health. In line with bioecological theory (Bronfenbrenner & Morris, 1998), both distal (e.g., COVID-related parental job changes, financial insecurity) and proximal risks (e.g., harsh discipline, parenting stress) have impacted child adjustment during the pandemic (Prime et al., 2020). Indeed, greater levels of children’s behavior problems were documented globally during the pandemic (Sari et al., 2022), and parental harsh discipline was linked to this increase in children’s behavior problems (Khoury et al., 2021). However, it is unclear how the effects of the COVID-19 pandemic on the link between harsh discipline and children’s behavior problems interact with the presence of other heightened familial risks in the context of the pandemic. Therefore, this study aimed to better understand the links between harsh discipline and children’s internalizing and externalizing problems during the pandemic by examining the dual moderating effects of parenting stress and negative COVID-19 pandemic impacts on these relations.

## Harsh Discipline and Child Behavior Problems

Harsh discipline practices include hostility, aggressive and emotionally charged verbal discipline (e.g., yelling or shouting), and physical punishment (e.g., spanking) (Chang et al., 2003). Harsh discipline is often considered an index of parents’ dysregulated reactions to child misbehavior expressed via overreactive or hostile behaviors (Deater-Deckard & Dodge, 1997). Overall, harsh discipline shows well-established associations with children’s behavior problems, broadly categorized into externalizing and internalizing problems. Externalizing problems often involve disruptive, antisocial, or aggressive behaviors, directed toward the external environment, while internalizing behaviors involve symptoms such as anxiety, depression, or somatic problems that impact the children’s internal psyche (Eisenberg et al., 2001). Harsh parenting shows strong associations with externalizing problems (Pinquart, 2017a) and predicts long-term adverse outcomes such as delinquency (Lansford et al., 2007) and depression (Wang & Kenny, 2014). Research has also documented positive links between harsh discipline and internalizing problems (Pinquart, 2017b), particularly in Western cultures, where children may be more likely to perceive harsh discipline as a sign of rejection, hostility, and lack of warmth and support from parents (Lansford et al., 2005).

Prior research also extensively documents the connections between harsh discipline and other familial risks, such as financial adversity, family conflict, parenting stress, and parents’ emotional and mental health difficulties (Conger & Conger, 2002). Notably, the COVID-19 pandemic, with its wide-ranging effects, introduced or heightened these risks for many families (Prime et al., 2020). Higher levels of parenting stress (Adams et al., 2021), more aggressive parent-child interactions (Humphreys et al., 2020; Muniz et al., 2019), and increased harsh discipline were reported during the pandemic (Sari et al., 2022). In a longitudinal study conducted in the United States during the COVID-19 pandemic, Khoury et al. (2021) found heightened positive associations between parental hostility and child externalizing problems. Given that multiple familial risk factors or stressors may exacerbate negative effects on child outcomes (Flouri & Midouhas, 2017), it is important to examine how multiple risk factors jointly influence children’s mental health in the context of the COVID-19 pandemic to better inform both basic research and interventions to ameliorate child mental health problems.

## Parenting Stress and Negative Impacts of the Pandemic

The COVID-19 pandemic severely impacted many individuals and disrupted various aspects of family life (Brown et al., 2020). From the early days of the pandemic, individuals faced the risk of infection, experienced illness or hospitalization, and encountered physical and emotional health problems (e.g., sleep difficulties and an increase in substance use). Social activities were reduced due to physical distancing and quarantine measures. Many parents faced job losses and reported economic hardships, disrupted and prolonged work schedules, and difficulties in home routines due to remote work during the pandemic (Prime et al., 2020). Children could not attend school, which increased parents’ responsibilities at home, and increased behavioral and emotional problems in children were reported (Deng et al., 2023). Although both mothers and fathers increased their parenting time during the pandemic, mothers reported feeling disproportionately responsible for managing childcare challenges and developmental activities, such as schooling, which are often less enjoyable for parents (Augustine et al., 2022, Douglas et al., 2024). The cumulative negative impacts across physical, social, emotional, and economic domains of family life, coupled with the disproportionate division of parenting labor, have been major risk factors for elevated parenting stress among mothers. These stressors have been linked to harsher discipline and disruptions in parent-child interactions (Neppl et al., 2016).

Parenting stress refers to the psychological stress on parents resulting from parenting demands, difficult child temperament and behavior, and negative parent-child interactions (Abidin, 1995). Elevated parenting stress indicates a higher discrepancy between a parent’s resources and supports and the demands of the parenting role (Deater-Deckard, 1998). School closures during the pandemic meant that children had to stay home more often, which forced many parents to work remotely while providing childcare (Prime et al., 2020). Moreover, lockdowns cut off children from needed daily activities such as play and peer socialization, further increasing parenting concerns (Lee et al., 2021). Parents also reported increases in stress due to concerns about communicating health-related issues to their children in a developmentally appropriate and sensitive way (Adams et al., 2021). Most caregivers also reported difficulties in obtaining social support and access to social services, mostly due to quarantine policies and isolation (Fontanesi et al., 2020). These increased levels of parenting stress did not return to pre-COVID-19 levels in the first two years of the pandemic (Adams et al., 2021).

Parenting stress is associated with both internalizing and externalizing problems in children (Crnic et al., 2005; Neece et al., 2012). Parents with elevated levels of parenting stress may have difficulties being emotionally available to their children and providing consistent care and support (Rose et al., 2018). Most studies have found that parenting stress is associated with nonoptimal parenting practices and harsh discipline (Jackson & Choi, 2018; Liu & Wang, 2015) and contributes to the transmission of harsh discipline practices across generations (Niu et al., 2018). Notably, research has shown that parents who engage in harsh discipline also perceive parenting as more stressful (Azar, 2002), suggesting a potential cycle where parenting stress and harsh discipline interact to increase the risk for children’s maladjustment. Thus, understanding the interaction between harsh discipline and parenting stress during the pandemic is crucial in assessing their dual impact on children’s behavior problems.

## Harsh Discipline, Parenting Stress, and Negative Impacts of the Pandemic

Harsh disciplinary practices often coincide with increased adversity in families and the lack of psychosocial resources to adaptively cope with hardships (Bøe et al., 2014; Conger et al., 1994). Children whose families experienced greater COVID-19 pandemic hardships were at higher risk for behavior problems (Gassman-Pines et al., 2020; Yoshikawa et al., 2020). Prior research suggests that mediational mechanisms linking negative pandemic impacts to children’s higher behavioral problems include increased parenting stress (Chung et al., 2022), parental worries about the pandemic (Waller et al., 2021), and increased hostile/harsh parental discipline (Chung et al., 2022; Khoury et al., 2021; Waller et al., 2021). However, there is limited empirical evidence regarding the interrelations or interaction effects of such risks.

Importantly, both harsh discipline and child behavior problems have been associated with other pandemic-related risk factors in prior research. For example, Chung et al. (2022) found that negative pandemic impacts increased parenting stress, subsequently leading to harsher parenting practices among mothers in Singapore. Similarly, punitive parenting reported by Turkish fathers during the pandemic predicted higher internalizing and externalizing problems in children, and this link was mediated by higher parenting stress (Ünsal & Acar, 2023).

A developmental psychopathology risk factor approach (Kazdin et al., 1997) contends that risks are reciprocally related and that the relation between risk factors and outcomes is conditional, such that the outcome depends on the timing, intensity, and degree of one or multiple risk factors in the child’s environment. Accordingly, a recent review argued that children’s mental health during the COVID-19 pandemic was impacted by the interaction of multiple risks that were either directly or indirectly related to children (Ng & Ng, 2022). Therefore, we argue that investigating the interplay of multiple risks as moderators of the link between harsh discipline and child outcomes can offer more information about their impact on children’s behavior problems in the pandemic context.

## Present Study

This study examined both proximal (harsh discipline and parenting stress) and distal risk factors (negative pandemic impacts) in relation to children’s internalizing and externalizing problems during the COVID-19 pandemic in a sample of mothers and children 2–6 years of age in the United States. First, we examined two two-way interaction effects: one between harsh discipline and parenting stress, and another between harsh discipline and the negative impacts of COVID-19. We expected that the positive relation between harsh discipline and children’s behavior problems would be stronger for families reporting higher levels of either parenting stress or negative COVID-19 pandemic impacts. Second, we examined whether there was a three-way interaction effect of harsh discipline, parenting stress, and negative pandemic impacts on children’s behavior problems. We expected that the positive association between harsh discipline and children’s behavior problems would be strongest when families reported both higher levels of parenting stress and higher negative pandemic impacts.

## Method

### Participants and Procedure

The study was approved by the university’s Institutional Review Board. Participants included biological mothers (*N* = 339) with at least one child between two and six years old (*M* = 49.68 months, *SD* = 13.05; 51.03% female). Roughly 28.10% of mothers (*M*_age_ = 35.37 years, *SD* = 7.76) had one child, 39.94% had two, 19.52% had three, and 12.42% had four or more children. Most mothers were married or living with a partner (82.01%). Roughly 83.68% of mothers identified as White or Caucasian, 8.90% Black or African American, 3.56% Asian or Asian American, 1.48% Native American, and 2.37% as of mixed race; 7.85% of mothers identified as Hispanic or Latino/Latina/Latinx ethnicity. Roughly half (51.32%) held bachelor’s degrees or higher, and median annual income was between $50,000 and $59,999.

Mothers were recruited for this remote, cross-sectional study via Amazon Mechanical Turk (MTurk) in Summer 2020 (*n* = 189) or Winter 2020 (*n* = 150). MTurk is an online platform where users complete paid human intelligence tasks (HITs) posted by social scientists, market researchers, and product developers. Specified subsets of users can be selected to view HIT ads, making MTurk a useful tool to collect data from participants remotely (Schleider & Weisz, 2015). Those whose MTurk accounts indicated they (1) identified as female, (2) were a parent, (3) resided in the United States, (4) had successfully completed at least 50 HITs, and (5) had a minimum 95% HIT approval rating could view the study advertisement. Interested mothers completed two additional eligibility questions in Qualtrics. Mothers who (6) reported fluency in written English and (7) had a two-to-six-year-old child progressed to an implied consent form. Those who provided implied consent began the questionnaire portion, which included questions about mothers, the target child, and parenting and lasted 40–45 min. Mothers also completed cognitive tasks by using Inquisit software version 6.5.1 (Millisecond Software, LLC) for approximately 10–15 min; this cognitive data was not utilized in the current study. Upon completion, mothers entered a payment code for $6.00 into MTurk for their participation.

### Measures

#### Harsh Discipline

Mothers reported their harsh discipline in the past year using the Parent-Child Conflict Tactics Scale (Straus et al., 1998). This scale was adapted in this study with permission from the authors such that wording of some items was slightly tailored to capture a broader range of harsh discipline behaviors, including some milder behaviors (e.g., Lightly slapped him/her on the hand, arm or leg), and consisted of 21 items assessing parent’s nonviolent discipline, psychological aggression, and physical assaults in the past year. Mothers’ responses were rated on a 7-point scale (ranging from 0 = *never occurred* to 6 = *more than 20 times*). Responses were recoded as frequency scores based on the midpoints for the response scale (i.e., 0 = 0, 1 = 1, 2 = 2, 3–5 times = 4, 6–10 times = 8, 11–20 times = 15, more than 20 times = 25), following the scoring protocol for this scale (Straus et al., 1998). To operationalize harsh discipline, psychological aggression (5 items, e.g., “Swore or cursed at him/her”) and physical assaults (7 items, e.g., “Threw or knocked him/her down”) subscales were analyzed. The frequency scores of psychological aggression and physical assaults were summed to represent harsh discipline levels, with higher scores reflecting higher levels of harsh discipline. Cronbach’s alpha for harsh discipline was 0.88.

#### Parenting Stress

Mothers reported their parenting stress using the Parenting Stress Index-Short Form (Abidin, 1995). Items were rated on a 4-point scale (1 = *strongly agree*, 5 = *strongly disagree*). Average scores of the parental distress (12 items, all reverse coded, e.g., “I feel trapped by my responsibilities as a parent”), parent-child dysfunctional interaction (12 items, mostly reverse coded, e.g., “My child smiles at me much less than I expected”), and difficult child (12 items, all reverse coded, e.g., “My child gets upset easily over the smallest things”) were computed to reflect the magnitude of mothers’ parenting stress, with higher scores reflecting higher levels of mothers’ parenting stress. Cronbach’s alpha for parenting stress was 0.96.

#### Negative Impacts of the Pandemic

The negative impacts of the COVID-19 pandemic were measured using the Epidemic-Pandemic Impacts Inventory (Grasso et al., 2020) as reported by mothers. In the present study, nine subscales that reflected negative impacts of the pandemic were used (i.e., work and employment, education and training, home life, social activities, economic, emotional health and well-being, physical health problems, physical distance and quarantine, infection history; 73 items in total; e.g., “Hard time making the transition to working from home”, “Unable to be with a close family member in critical condition”). For each item, mothers indicated whether the hardship did not happen or did not apply (score of 0), applied to either themselves or someone else in their home (1), or applied to both them and someone else in their home (2). Item-level scores were summed for each subscale, and nine subscales that reflected negative impact were summed to create the score reflecting the negative impacts of the pandemic. The inventory showed good validity among adults in the US (Grasso et al., 2021). Cronbach’s alpha in this study was 0.95.

#### Child Internalizing and Externalizing Problems

Child internalizing and externalizing problems were measured using the Child Behavior Checklist (Achenbach & Rescorla, 2000). Mothers rated their children’s behaviors during the past six months using a 3-point scale (ranging from 0 = *not true* to 2 = *very/often true*). Sum scores were computed for each subscale, with higher scores representing more internalizing (36 items, e.g., “Gets too upset when separated from parents”) or externalizing (24 items, e.g., “Cannot sit still, restless, or hyperactive”) behaviors. Cronbach’s alphas were 0.93 for the externalizing subscale and 0.96 for the internalizing subscale. Seven and a half percent and 22.0% of children had symptoms above the clinical cutoffs (*t*-scores > = 64) for externalizing or internalizing problems, respectively (Achenbach & Rescorla, 2000).

### Data Analytic Plan

We tested the moderating effects of parenting stress, negative pandemic impacts, and their dual moderating effects on the links between harsh discipline and child internalizing and externalizing problems (respectively) by using the PROCESS macro (model 3; Hayes, 2013) in R software (version 4.3.1). For models with either internalizing and externalizing problems as outcomes, all variables of interest in the models had linear relations with outcomes, and there were no multicollinearity issues between predictors (Variance Influence Factors (VIF) for each independent predictor < 3). The normal distribution and linearity of residuals assumptions of regression models were met but the homoscedasticity assumption was violated. As recommended by Regorz (2021), the option for heteroskedasticity-consistent standard errors (HC4) was used while running analyses to correct errors for heteroskedasticity.

There were only three missing values for one of the predictors at the scale level (i.e., harsh parenting, negative pandemic impacts, or parenting stress) out of 339 families. A test of Missing Completely at Random (MCAR; Little, 1988) confirmed that the missingness was at random, χ^2^(12) = 18.39, *p* = 0.10. The PROCESS macro handled missing data with listwise deletion by default, and three cases were omitted. Internalizing and externalizing problems were tested in separate models, and the total analytic sample was *N* = 336 for both internalizing and externalizing problems. Harsh discipline, parenting stress, and the negative impacts of the pandemic were included in the model. All continuous predictors were mean centered automatically by the PROCESS macro, and all possible interaction effects were estimated. To analyze the pattern of significant two-way interactions, simple slope analyses were conducted based on high, medium, and low values of the moderators (i.e., negative pandemic impact and parenting stress) which corresponded to 1 standard deviation (*SD*) above the mean, at the mean, and 1 *SD* below the mean. To analyze significant three-way interactions, tests of simple slopes and slope differences were conducted at low (1 *SD* below mean) and high (1 *SD* above mean) values of the moderators (Dawson & Richter, 2006).

## Results

### Preliminary Analyses

Descriptive statistics and correlations are reported in Table 1. Children’s internalizing and externalizing problems reported by parents were significantly and positively correlated. Harsh discipline, parenting stress, and negative pandemic impacts were all significantly and positively correlated with internalizing and externalizing problems. Higher family income (*r* = −0.14, *p* < 0.05) had a modest inverse correlation with externalizing problems but was not correlated with internalizing problems. There were no significant differences in mothers’ reported parenting stress, harsh parenting, or the negative impacts of the pandemic by mother-reported race or ethnicity (all *p*s > 0.05). Child age and gender were not correlated with any study variables. Since correlations between sociodemographic variables and outcomes were only modestly correlated or not correlated, we did not include these variables as covariates in our primary analyses.

**Table 1:** Descriptive data of study variables

Variable	1	2	3	4	5	*M*	*SD*	Min	Max
1. Harsh Discipline	1	0.55**	0.46**	0.61**	0.61**	34.38	44.68	0	233
2. Parenting Stress		1	0.55**	0.75**	0.71**	23.89	9.57	0	82
3. Negative Pandemic Impacts			1	0.60**	0.52**	23.75	16.51	12	52
4. Internalizing Problems				1	0.77**	10.82	12.75	0	58
5. Externalizing Problems					1	12.37	9.31	0	41

**p < 0.01.

### Internalizing Problems Model

**Fig. 1:**
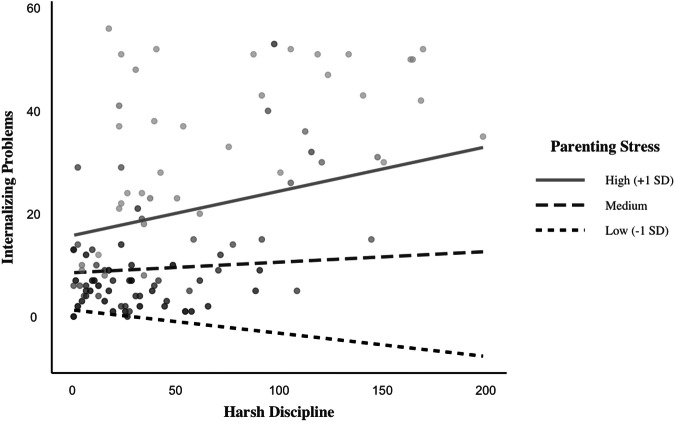
Two-way interaction between harsh discipline and parenting stress effects on internalizing problems

The model including the predictors of harsh discipline, parenting stress, negative pandemic impacts, and all interaction terms was significant and explained 71% of the variance in children’s internalizing problems, *F*(7, 328) = 116.21, *p* < 0.001. As shown in Table 2, with respect to two-way interaction effects, parenting stress moderated the relation between harsh discipline and internalizing problems, *b* = 0.0069, *t*(328) = 2.83, *p* < 0.001, 95% CI [0.0021, 0.0117]. Figure 1 shows the simple slopes at low, medium, and high levels of parenting stress. For children whose parents reported higher degrees of parenting stress, harsh discipline was positively associated with internalizing problems, *b* = 0.0863, *t* = 3.25, *p* < 0.001. For children whose parents reported either moderate or lower degrees of parenting stress, these relations were not significant, *p* = *0*.286 and *p* = 0.132, respectively. In contrast to our hypothesis, there was not a significant two-way interaction between harsh discipline and negative impacts of the pandemic on children’s internalizing problems, *b* = −0.0003, *t*(328) = −0.20, *p* = 0.843.

**Table 2:** The effects of harsh discipline, parenting stress, negative pandemic impacts on internalizing and externalizing problems

	Internalizing Problems				Externalizing Problems			
	*β*	*b*	*SE(HC4)*	*t*	*β*	*b*	*SE(HC4)*	*t*
Constant		8.5214	0.5331	15.98***		12.0431	0.4375	27.52***
Predictors								
Harsh Discipline	0.0720	0.0205	0.0194	1.06	0.3324	0.0693	0.0142	4.87***
Parenting Stress	0.5701	0.7599	0.0993	7.65***	0.4915	0.4783	0.0652	7.34***
Negative Pandemic Impacts	0.1163	0.0898	0.0335	2.53*	0.0814	0.0459	0.032	1.43
Harsh Discipline x Parenting Stress	0.2314	0.0069	0.0024	2.83**	0.0652	0.0014	0.0014	0.99
Harsh Discipline x Pandemic Impacts	−0.0150	−0.0003	0.0013	−0.2	−0.1762	−0.0022	0.0009	−2.39*
Parenting Stress x Pandemic Impacts	0.1714	0.0138	0.0048	2.91**	0.1411	0.0083	0.0030	2.76**
Harsh Discipline x Parenting Stress x Pandemic Impacts	−0.0724	−0.0001	0.0001	−2.37*	−0.0082	−0.0000	0.0000	−0.24

^a^HC4 refers to heteroscedasticity consistent standard errors

*p < 0.05; **p < 0.01, ***p < 0.001

**Fig. 2:**
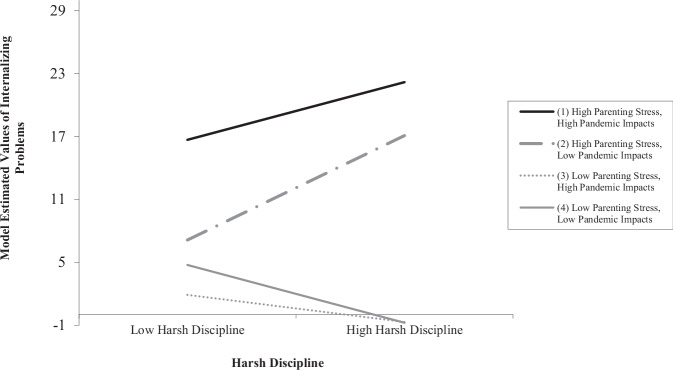
Three-way interaction between harsh discipline, parenting stress, and negative pandemic impacts on internalizing problems

Results supported our main hypothesis for a three-way interaction effect, such that parenting stress and negative impacts of the pandemic significantly and jointly moderated the relation between harsh discipline and internalizing problems, *b* = −0.0001, *t*(328) = −2.37, *p* = 0.018, 95% CI [−0.0002, −0.0000] (Table 2). Simple slope tests indicated that higher levels of harsh discipline were associated with more internalizing problems when parents reported higher parenting stress and higher negative impacts of the pandemic, *b* = 0.061, *t* = 4.78, *p* < 0.001. In contrast, higher levels of harsh discipline were not associated with more internalizing problems when parents reported lower parenting stress and lower negative impacts of the pandemic, *b* = −0.062, *t* = −1.85, *p* = 0.066. When parenting stress was higher and pandemic impacts were lower, this combination also yielded significant positive effects of harsh discipline on internalizing problems, *b* = 0.111, *t* = 4.78, *p* < 0.001. However, the combination of lower parenting stress and higher negative pandemic impacts was not significant, *b* = −0.029, *t* = −0.94, *p* = 0.347. The summarized results are presented in Fig. 2.

To examine the difference in slopes, post hoc analyses were conducted (Dawson & Richter, 2006). Simple slopes tests revealed a significant difference between slope (1), representing parents reporting higher parenting stress and higher negative COVID-19 impacts, and slope (2), representing parents reporting higher parenting stress and lower negative COVID-19 impacts, *t* = −2.02, *p* = 0.044. Though both showed an exacerbating effect, there was a stronger relation between harsh discipline and internalizing problems when parents reported higher parenting stress and lower (as compared to higher) impacts of the pandemic. As we expected, slope (1), representing parents’ reporting higher parenting stress and higher negative COVID-19 impact, was steeper than both slope (3) (lower parenting stress, higher COVID-19 impact; *t* = 3.50, *p* < 0.001) and slope (4) (lower parenting stress, lower COVID-19 impact; *t* = 3.23, *p* < 0.001).

### Externalizing Problems Model

**Fig. 3:**
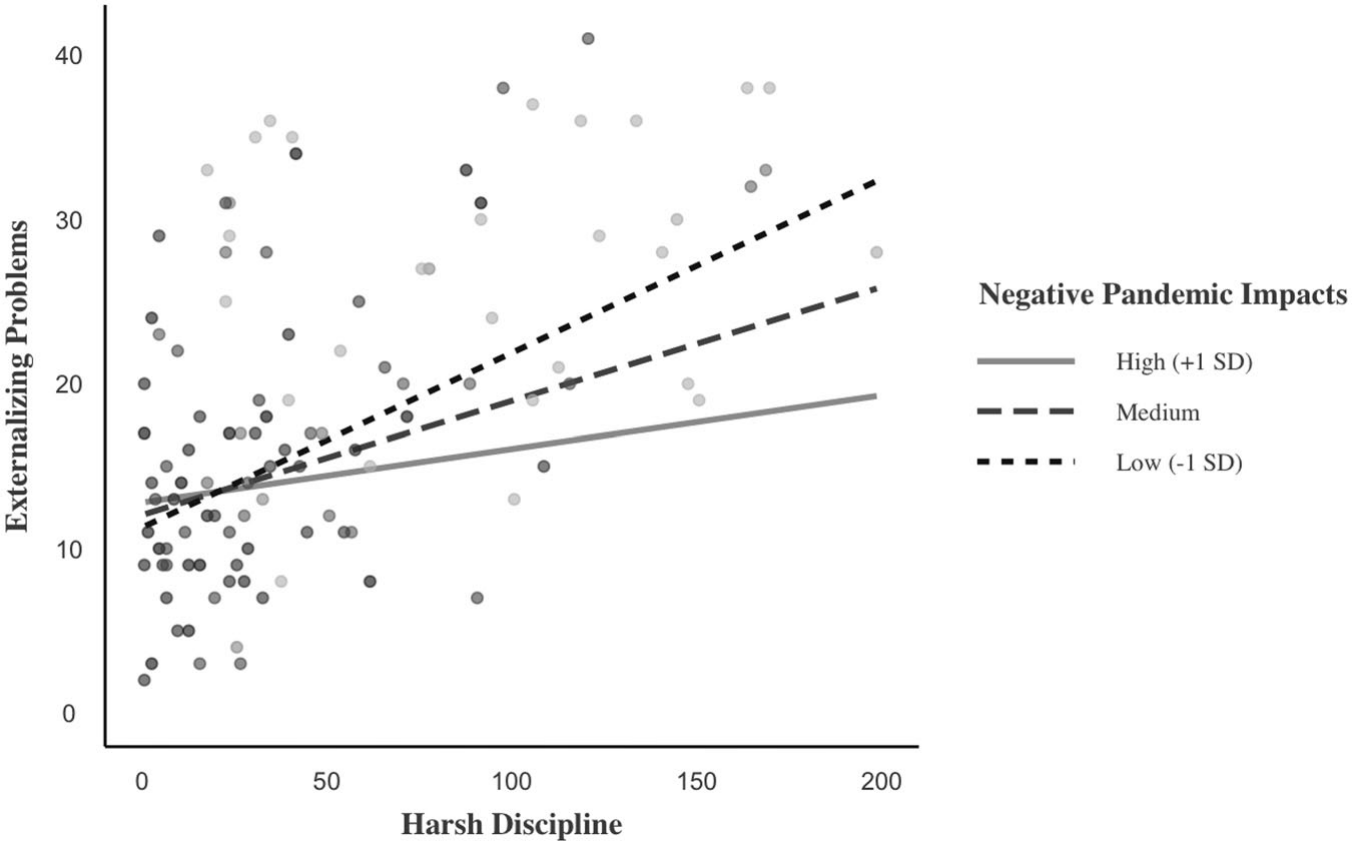
Two-way interaction between harsh discipline and negative pandemic impacts on externalizing problems

The model including all predictors such as harsh discipline, negative pandemic impacts, and parenting stress was significant and explained 59% of the variance in children’s externalizing problems, *F*(7, 328) = 62.19, *p* < 0.001. As far as two-way interaction effects, the negative impacts of the pandemic moderated the link between harsh discipline and children’s externalizing problems, *b* = −0.002, *t*(328) = −2.39, *p* = 0.017, 95% CI [−0.0041, −0.0004]. Figure 3 shows the simple slopes at low, medium, and high levels of negative impacts of the pandemic. The association between harsh discipline and children’s externalizing problems was weaker in families reporting higher levels of pandemic impacts (*b* = 0.0325, *t* = 1.88, *p* = 0.061) compared to those reporting moderate (*b* = 0.069, *t* = 4.94, *p* < 0.001) or lower levels of these impacts (*b* = 0.1060, *t* = 4.67, *p* < 0.001). In contrast to the model of internalizing problems, parenting stress did not moderate the relation between harsh discipline and children’s externalizing problems, *b* = 0.0014, *t*(328) = 0.99, *p* = 0.319. Also, in contrast to our hypothesis, the three-way interaction between harsh discipline, parenting stress, and negative pandemic impacts was not significant in predicting children’s externalizing problems, *b* = 0.000*, t*(328) = −0.24, *p* = 0.808. However, results indicated main effects of both harsh discipline, *b* = 0.069, *t*(328) = 4.87, *p* < 0.001, 95% CI [0.0413, 0.0972], and parenting stress, *b* = 0.4783, *t*(328) = 7.34, *p* < 0.001, 95% CI [0.3501, 0.6064], on children’s externalizing problems.

### Post-hoc Analyses

Considering the significant three-way interaction results for children’s internalizing problems as shown in Fig. 2, we conducted post-hoc analyses to test whether mean levels of internalizing problems would differ among the resulting groups characterized by varying levels of parenting stress and negative impacts of the pandemic. Given that subgroup sample sizes were too small when grouping data based on one standard deviation below/above the mean, we applied a median split method. Using this method, continuous variables (harsh discipline, parenting stress, negative pandemic impacts) were categorized into ‘high’ and ‘low’ groups. Pairwise comparisons in an ANOVA test revealed that in families reporting higher levels of harsh discipline, parenting stress, and negative pandemic impacts, children had the highest mean levels of internalizing problems in this group (*M* = 23.32, *SE* = 1.13) compared to all other groups, *p*s < 0.001. In contrast, other groups showed comparatively lower levels of internalizing problems: those experiencing higher harsh discipline, higher parenting stress, and lower negative pandemic impacts (*M* = 12.97, *SE* = 1.61); higher harsh discipline, lower parenting stress, and higher negative pandemic impacts (*M* = 4.60, *SE* = 2.28); and higher harsh discipline, lower parenting stress, and lower negative pandemic impacts (*M* = 4.55, *SE* = 1.70). Importantly, these analyses indicated that children from families where harsh discipline, parenting stress, and negative pandemic impacts were reported at the highest levels showed an average score of 23 on the internalizing problems scale. This corresponds to a t-score of 68, which is above the clinical cut-off for an internalizing disorder on the CBCL (t-score ≥ 64) as established by Achenbach & Rescorla (2000). This finding suggests that a higher accumulation of risks in a child’s familial environment was a particular concern for children’s internalizing problems during the pandemic.

## Discussion

The present study sought to examine whether parenting stress and the negative impact of the pandemic exacerbated the links between harsh discipline and children’s internalizing and externalizing problems during the COVID-19 pandemic. Children in this study had the highest levels of internalizing problems when all risk factors, namely harsh discipline, parenting stress, and negative COVID-19 impacts, were higher. Moreover, higher degrees of parenting stress exacerbated the positive link between harsh discipline and internalizing problems, both for lower and higher degrees of negative pandemic impacts as reported by mothers. Thus, findings supported our hypotheses that children’s internalizing problems during the pandemic would be affected by the interaction of multiple risks, indexed by harsh discipline, parenting stress, and negative pandemic impacts, and that parenting stress would exacerbate the effects of harsh parenting on children’s internalizing problems.

For externalizing problems, in contrast, pandemic impacts did not exacerbate the existing harmful effects of harsh parenting as hypothesized; rather, this association weakened as families reported increasing levels of pandemic impacts. However, this finding can be explained from a dynamic systems theory perspective that suggests that recurrent, stable patterns in parent-child interactions (e.g., coupling between harsh discipline and external behavior problems) can become destabilized when strong, abrupt external perturbations introduce disturbances to the family system (Granic & Patterson, 2006). This might be especially true in the context of the COVID-19 pandemic where its course and effects were unpredictable, and multiple elements of the family system (e.g., parenting behaviors, parenting stress, parent-child discipline interactions) were likely to be impacted. Accordingly, our findings highlight the importance of grounding work in the theoretical context of dynamic and family systems theories when examining the impacts of major external stressors such as a pandemic on the interactive effects of familial proximal and distal risks on children.

### Three-way Interaction Effects on Children’s Internalizing Problems

A significant three-way interaction suggested that the negative impacts of the pandemic moderated the interaction between harsh discipline and parenting stress when predicting children’s internalizing problems. Children showed the highest levels of internalizing problems when all three risks (harsh discipline, parenting stress, and negative pandemic impacts) were present in their environments at higher degrees. Although pandemic impacts moderated the relation regardless, the link between harsh discipline and children’s internalizing problems was statistically somewhat stronger, even though mean levels of internalizing were lower, in families reporting higher levels of parenting stress with lower (compared to higher) negative impacts of COVID-19 (see Fig. 2). Complex interactive processes between these variables may be the reason for this finding. It is possible that when both parenting stress and the negative impact of COVID-19 are at higher degrees, parenting stress may carry more relative weight in the effects of harsh discipline on children’s internalizing problems due to it being a more proximal risk factor, particularly when negative pandemic impacts are less pronounced. In the context of the pandemic, parents reported an increase in childcare responsibilities due to homeschooling children and difficulties in balancing childcare and work (Brown et al., 2020), which might co-occur with increased parenting stress and lead to its interactive impacts (with harsh discipline) on children’s mental health.

Research has shown that factors within the parent-child subsystem – such as inconsistent discipline, greater hostility, fewer routines, and less parental supportiveness – were particularly concerning for children’s behavior problems during the pandemic (Prime et al., 2020). In line with the family stress model (Conger & Conger, 2002), children growing up in households with elevated proximal and/or distal risks during COVID-19 were more likely to be exposed to harsh parenting. Harsh parenting, characterized by parents’ emotional dysregulation and aggressive physical and emotional parenting behaviors (Chang et al., 2003), hinders the child’s ability to learn effective regulation strategies from parents. Furthermore, exposure to parental negativity, threats, and hostility can disrupt the child’s sense of emotional security within the family (Davies & Cummings, 1994), potentially leading to symptoms of emotional distress, fearfulness, and anxiety. Our findings suggest that the interaction between harsh discipline, parenting stress, and the negative impacts of the pandemic might be associated with children’s internalizing problems through disruptions in affective quality in parent-child relationships.

### Two-Way Interaction Effects on Children’s Internalizing Problems

Higher parenting stress was an important risk factor and exacerbated the relation between harsh discipline and children’s internalizing problems. Internalizing problems (e.g., anxiety, fear, withdrawal) reflect the internal emotional states of children; therefore, these symptoms might have been more likely to be impacted by parenting stress and the quality of the parent-child relationship compared to externalizing problems. Costa et al. (2006) found that dysfunctional parent-child interactions (a factor from the Parenting Stress Index; Abidin, 1995) were only associated with internalizing but not externalizing problems in children aged 5–17 years.

Parents experiencing higher parenting stress may be inadvertently more overreactive (Guajardo et al., 2009) and less warm (Qian et al., 2023) to their children, which may intensify the negative impact of harsh discipline practices on children’s internalizing symptoms. Highly stressed parents may also perceive their interactions with children as more challenging or as having lower emotional quality (Costa et al., 2006), and they might be less sensitive to their children (Pereira et al., 2012). Consequently, children in our sample who experienced harsh discipline may have also received lower emotional support/warmth from parents, which could have worsened their internalizing problems.

Previous work has shown that exposure to uncontrollable, large-scale disasters may cause ongoing stress and a sense of helplessness in children, often associated with symptoms such as depression, panic, and anxiety (Rubens et al., 2018; Fischer et al., 2023). A meta-analysis found that the prevalence rates of depression, anxiety symptoms, and sleep disturbances among youth were higher during the pandemic compared to pre-pandemic levels (Deng et al., 2023). Factors like the fear of the virus itself, uncertainties about the progress of the pandemic, and the increased worry and anxiety that COVID-19 caused in both children and parents might have contributed to our findings specific to internalizing problems.

For parents reporting medium or lower degrees of parenting stress, there was no significant relation between harsh discipline and children’s internalizing problems. This result may be partially related to the moderate positive correlation between parenting stress and harsh discipline in our sample (*r* = 0.55). Previous studies showed that lower levels of parenting stress are associated with more optimal parenting, such as high emotional warmth and parental monitoring (Bonds et al., 2002). As a result, there might not be a link between harsh disciplinary methods and internalizing problems for some children in our sample if less stressed parents are using more optimal parenting practices overall.

Contrary to our hypothesis, we did not find a significant two-way interaction between harsh discipline and pandemic impacts on internalizing problems. As previously noted, parenting stress, as a more proximal risk factor, seemed more directly linked to children’s mental health regardless of the extent of a family’s exposure to the negative impacts of the pandemic.

### Three-Way Interaction Effects on Children’s Externalizing Problems

Unexpectedly, the interactive effects of parenting stress and negative pandemic impacts did not moderate the link between harsh discipline and externalizing problems. Harsh discipline remained a significant positive predictor of children’s externalizing problems when all other interaction effects were present in the model. Notably, harsh and coercive discipline has been highlighted as the primary concern for children’s mental health during the pandemic (Prime et al., 2020). However, Penner et al. (2022) similarly found a direct link between parenting variables (e.g., inconsistent discipline, greater hostility) and children’s internalizing and externalizing problems yet did not find an effect of COVID-19-stressors on child mental health when other variables (parental depression/anxiety, parental hostility) were considered. Although not measured in our study, the negative impacts of COVID-19 could potentially have contributed to an increase in harsh discipline and parenting stress. Consequently, when considering the effects of parental variables such as harsh discipline and parenting stress, the direct impact of negative pandemic effects on a child’s mental health might be comparatively weaker.

### Two-Way Interaction Effects on Children’s Externalizing Problems

There was a significant two-way interaction between harsh discipline and the negative impact of the pandemic on externalizing problems such that the relation between harsh discipline and externalizing problems weakened for families reporting higher levels of negative impacts of the pandemic. While this finding may seem initially counterintuitive given prior research demonstrating well-established positive links between harsh parenting and child externalizing problems, the unexpected, overarching changes in the family system during the pandemic need to be considered while interpreting these effects.

A dynamic systems perspective suggests that well-established, stable links between parent and child behaviors may be weakened or even disrupted when novel, acute environmental stressors impact the family system, destabilizing ongoing interaction patterns (Fraley & Brumbaugh, 2004; Granic & Patterson, 2006). As parents become preoccupied with these external stressors, their interactions with children may become less predictable or stable. For example, some parents may acquiesce more frequently to children’s misbehavior because they lack the energy or resources to engage in challenging interactions with their children, whereas others may become more rigid in their disciplinary limits, punishing children more immediately. Accordingly, this unpredictability may weaken the coupling between harsh discipline and child externalizing problems while families are dealing with broader pandemic-related adversities. It is also possible that as parents become overwhelmed by bigger pandemic-related stressors (e.g., financial insecurity, health concerns), they prioritize managing these external stressors, which reduces their attention towards their children. Pandemic-related negative impacts may also reduce parents’ monitoring of their children’s problem behaviors, potentially leading to lower reports of externalizing behaviors. Previous work has shown lower levels of parents’ monitoring in parents with higher sociodemographic risks (Kotchick et al., 2005). Future research could examine whether the cumulative effects of harsh parenting and pandemic-related stress are linked to lower reports of externalizing problems through their association with reduced parental monitoring as a potential mediator.

As previous research suggests, harsh parenting disrupts a child’s emotional security within the parent-child relationship (Chang et al., 2003). In the context of heightened stress and uncertainty during COVID-19, children exposed to higher levels of harsh parenting may have exhibited more inhibited responses rather than acting out (Crittenden & DiLalla, 1988; Koenig et al., 2000). Our findings indicate that the combined effects of harsh parenting and parenting stress increased children’s internalizing symptoms, which may have indirectly suppressed externalizing behaviors if children felt insecure with their parents and feared potential consequences of misbehaviors.

Parenting stress had a positive association with children’s externalizing problems, but, contrary to our expectations, it did not moderate the link between harsh discipline and externalizing problems. This result is aligned with other studies showing that parenting stress relates directly to children’s externalizing problems (Anthony et al., 2005) as well as total behavior problems (sum of internalizing and externalizing problems) (Crnic et al., 2005; Neece et al., 2012). The lack of an interaction effect might also suggest that the link between harsh discipline and externalizing problems is already robust on its own, thus the influence of parenting stress is weaker in comparison.

It is possible that parenting stress mediates rather than moderates the relation between parents’ overall psychosocial stress and child adjustment, as elevated parenting stress often disrupts supportive and positive discipline and increases harsh discipline. Indeed, previous work found the link between increased parenting stress during the COVID-19 pandemic and greater harsh discipline, mediated through decreased parent-child relationship quality (Chung et al., 2022). Mediation models can also test whether the negative impacts of the pandemic and harsh parenting are linked to higher internalizing/externalizing behaviors in children, which may, in turn, predict higher parenting stress. These models help differentiate parent-driven vs. child-driven effects, aligning with longitudinal studies suggesting that relations between behavior problems and parenting stress are bidirectional (Kochanova et al., 2022). However, given the cross-sectional nature of the current study, mediational models are limited to interpret the directionality of these potential associations.

### Limitations, Future Directions, and Implications

Several limitations should be considered when interpreting these findings. First, all study variables were collected exclusively through maternal self-report questionnaires during the COVID-19 pandemic. This common method variance is a potential limitation that could have inflated the relations between predictors and child outcomes. Parents who report more conflicts with their children may also report more negatively about children’s behaviors (Stokes et al., 2011), which suggests a potential bias in relying solely on parental reports in examining child behavior problems. However, many of our research questions required the use of self-report measures because parenting stress and negative impacts of the pandemic are subjective and reflect parents’ experiences of stress in their parenting role and the perceived negative impacts of the pandemic. Also, direct observations of harsh discipline in research are rare, thus research tends to rely on parental-self reports of harsh discipline. Moreover, due to pandemic restrictions, in-person observational data collection was not feasible, which led us to utilize online questionnaires. Despite these limitations, our regression models revealed no issues of multicollinearity, suggesting that predictor variables were not highly intercorrelated and could independently contribute to the outcomes. Future studies on children’s behavior problems might benefit from utilizing multiple informants when relying on self-report measures, and from incorporating both lab and home observations. Second, we measured the global negative impacts of the pandemic on families using sum scores from the negative subscales of the Epidemic-Pandemic Impacts Inventory (EPII; Grasso et al., 2020). Although this approach is valuable for understanding the cumulative impact of risks, testing how risks in specific areas (e.g., physical health, social, emotional, and economic domains) differed in impact and differed by familial sociodemographic factors could offer more information about which types of risks are uniquely associated with internalizing or externalizing child behavior problems for various families.

Third, participants were sampled through Amazon Mechanical Turk and our sample only included biological mothers. Previous research suggests that mothers were disproportionately affected by increased caregiving demands during the pandemic (Augustine et al., 2022; Douglas et al., 2024). However, including secondary caregivers could provide a more comprehensive understanding of the division of labor between parents during the pandemic and the degree of spousal support, and how these factors impact parenting stress, harsh parenting, and child behavior problems.

Fourth, while MTurk is commonly used for social science research (Paolacci & Chandler, 2014), there are limitations to its representativeness. Data collected through MTurk may not fully represent the broader population due to underrepresentation of racial and ethnic minority groups, as well as the tendency for online participants to be younger and better educated than the general U.S. population. In this study, half of the mothers had education levels equal to or higher than a bachelor’s degree, which limits our ability to generalize the findings to parents with lower levels of education.

Fifth, the cross-sectional design of this study limits our ability to address causal mechanisms and make temporal predictions about child outcomes. Without longitudinal data, we cannot assess how harsh discipline and parenting stress changed during the pandemic or how changes in these risk factors interacted over time. Additionally, child behavior problems were measured only during the pandemic, not before, which prevented us from capturing pre-pandemic levels of behavior problems in our sample. It is possible that children whose parents reported higher behavior problems during the pandemic also had higher levels of these problems pre-pandemic, potentially influenced by pre-pandemic levels of harsh discipline and parenting stress.

Although parents across racial groups did not significantly differ in any of the study variables (the negative impacts of the pandemic, parenting stress, harsh parenting) in this study, previous research has shown that Black and Latinx children, especially those from low-income families, disproportionately experienced the negative impacts of the COVID-19 pandemic, marked by higher economic stress and an increased likelihood of health concerns (Bogan et al., 2022; Vargas et al., 2020). Despite these increased risks, research also suggests that Black and Latinx families benefit from rich cultural practices, resources, and strengths that may help them cope with these stressors (Causadias & Neblett, 2024). Future research can explore individual competencies and familial coping strategies within various ethnic and racial groups to better understand culturally relevant resilience processes in the context of major global stressors such as a pandemic.

There is also a need to examine the complex links between parenting and child outcomes during a global pandemic across countries or cultures. Variations across countries or cultures may impact parenting approaches and families’ responses to stress, which may, in turn, affect child behavior outcomes. For example, a study including mothers from China, the Netherlands, and Italy found that harsh parenting was a global phenomenon in all countries during the COVID-19 pandemic, predicted by marital conflict and maternal psychopathology (Riem et al., 2021). Yet, there were unique risk and protective factors in each country, for instance, grandmothers’ support of mothers with younger children particularly lowered the risk for harsh parenting in China. Future research should explore these cultural dimensions to provide more nuanced insights into the global effects of pandemics on child development.

Despite the limitations of this study, our findings may offer important new insights into the interactive effects of multiple risks during pandemics and might suggest implications for intervention researchers. Selecting interventions that focus on assisting parents in managing their stress and enhancing interactions with their children may be particularly helpful for families heavily affected by the negative impacts of pandemics. Evidence-based behavioral parenting interventions, such as Parent-Child Intervention Therapy (PCIT; Eyberg, 1988), Parent Management Training (PMT; Kazdin, 1997), or the Triple-P-Positive Parenting program (Sanders, 2012) show promise in improving parental support, mitigating harsh discipline, and addressing children’s behavioral problems. Both the TripleP program and PCIT have also demonstrated effectiveness in reducing parenting stress (Thomas & Zimmer-Gembeck, 2007). These programs could be recommended for families challenged by difficult child behaviors during a pandemic or post-pandemic. Cognitive Behavioral Stress Management programs, which aim to enhance participants’ stress management skills through adaptive coping strategies, relaxation techniques, and meditation, have also been found effective in reducing the stress and physical and emotional exhaustion resulting from the parenting role (Urbanowicz et al., 2023).

### Summary

The current study enhanced our understanding of the interactive effects of multiple risks (harsh discipline, parenting stress, and negative impacts of the pandemic) on children’s internalizing and externalizing problems during the COVID-19 pandemic. Children whose parents reported higher levels of harsh discipline, parenting stress, and negative pandemic impacts were at greater risk of internalizing problems. Parenting stress seems to have been a prominent factor in children’s mental health during the COVID-19 pandemic, strengthening the positive link between harsh discipline and children’s internalizing problems. Our findings align with the ecological perspective (Bronfenbrenner & Morris, 1998) and suggest that proximal risks such as harsh discipline and parenting stress more directly impact children’s behavior problems compared to more distal familial risks. Our findings underscore the need to address harsh discipline and parenting stress in family interventions, considering that they heighten children’s behavior problems in the context of major stressors such as a global pandemic.
